# Bayesian evidence synthesis to estimate subnational TB incidence: An application in Brazil

**DOI:** 10.1016/j.epidem.2021.100443

**Published:** 2021-02-20

**Authors:** Melanie H. Chitwood, Daniele M. Pelissari, Gabriela Drummond Marques da Silva, Patricia Bartholomay, Marli Souza Rocha, Mauro Sanchez, Denise Arakaki-Sanchez, Philippe Glaziou, Ted Cohen, Marcia C. Castro, Nicolas A. Menzies

**Affiliations:** a Department of Epidemiology of Microbial Diseases, Yale School of Public Health, 60 College Street, New Haven CT 06510 United States; b Chronic and Airborne Disease Surveillance Coordination, Ministry of Health, SRTVN Qd. 701, Via W5 Norte, Lote D, Ed. PO 700, Brasília Brazil; c Department of Tropical Medicine, University of Brasília, Campus Universitário Darcy Ribeiro, s/n Asa Norte, Brasília Brazil; d World Health Organization, Avenue Appia 20, Geneva Switzerland; e Department of Global Health and Population, Harvard T.H Chan School of Public Health, 677 Huntington Ave, Boston MA, 02115 United States

**Keywords:** Tuberculosis, Bayesian, Subnational, Estimation, Incidence

## Abstract

**Background:**

Evidence on local disease burden and the completeness of case detection represent important information for TB control programs. We present a new method for estimating subnational TB incidence and the fraction of individuals with incident TB who are diagnosed and treated in Brazil.

**Methods:**

We compiled data on TB notifications and TB-related mortality in Brazil and specified an analytic model approximating incidence as the number of individuals exiting untreated active disease (sum of treatment initiation, death before treatment, and self-cure). We employed a Bayesian inference approach to synthesize data and adjust for known sources of bias. We estimated TB incidence and the fraction of cases treated, for each Brazilian state and the Federal District over 2008–2017.

**Findings:**

For 2017, TB incidence was estimated as 41.5 (95 % interval: 40.7, 42.5) per 100 000 nationally, and ranged from 11.7–88.3 per 100 000 across states. The fraction of cases treated was estimated as 91.9 % (89.6 %, 93.7 %) nationally and ranged 86.0 %–94.8 % across states, with an estimated 6.9 (5.3, 9.2) thousand cases going untreated in 2017. Over 2008–2017, incidence declined at an average annual rate of 1.4 % (1.1 %, 1.9 %) nationally, and −1.1%–4.2 % across states. Over this period there was a 0.5 % (0.2 %, 0.9 %) average annual increase in the fraction of incident TB cases treated.

**Interpretation:**

Time-series estimates of TB burden and the fraction of cases treated can be derived from routinely-collected data and used to understand variation in TB outcomes and trends.

## 1. Introduction

Globally, an estimated 40 % of incident tuberculosis (TB) cases are not diagnosed, and many individuals with active TB who are missing from official case reports likely never receive treatment (Chin and Hanson, 2017). Undetected and untreated TB is a major public health problem, as these individuals face high mortality risks in the absence of appropriate treatment (Tiemersma et al., 2011), and continue to infect others in their households and communities (Dharmadhikari et al., 2014). Shortening the time between symptom appearance and treatment initiation is important both to reduce morbidity and mortality and to interrupt TB transmission (Churchyard et al., 2017).

Efforts to strengthen TB case detection typically prioritize geographic areas with higher disease burden. However, without special studies that confirm burden estimates (World Health Organization, 2010) or verify the strength of case detection (Glaziou et al., 2012), unobserved variation in the fraction of TB cases that are identified will systematically distort TB burden estimates. In this context, a lower TB notification rate could indicate lower disease incidence, or alternatively signal incomplete case detection, with these two explanations having conflicting implications for program management. Therefore, understanding variation in the completeness of case detection over both time and space can provide tuberculosis control programs with information about subnational differences and trends in TB incidence and service coverage, enabling a distribution of TB control resources that is responsive to local needs.

Brazil has been identified by the World Health Organization (WHO) as one of thirty high TB burden countries, with 79 222 new and relapse cases reported in 2017 (World Health Organization, 2018). Within Brazil, TB notification rates follow clear regional and socioeconomic patterns (Barreto et al., 2011; Harling et al., 2017), with specific populations—such as indigenous peoples and incarcerated individuals—exhibiting higher rates of TB than the general population (Basta et al., 2005; Carbone et al., 2015). These factors are likely to contribute to differences in TB incidence and case reporting rates across Brazilian states. Consequently, a national case detection estimate, which the WHO currently produces annually, is of limited utility for local tuberculosis programs, given the country’s large size and epidemiological heterogeneity. State-level estimates, in contrast, would capture a good deal of subnational heterogeneity in tuberculosis burden and, given Brazil’s decentralized healthcare system, be of value to decision makers.

In this analysis, we present a new method to estimate subnational TB incidence and the fraction of individuals who initiate treatment. Using routinely collected data, we present estimates of TB incidence rates and the fraction of cases initiating treatment for the 26 Brazilian states and the Federal District from 2008–2017.

## 2. Methods

### 2.1. Estimation strategy

**Fig. 1.**
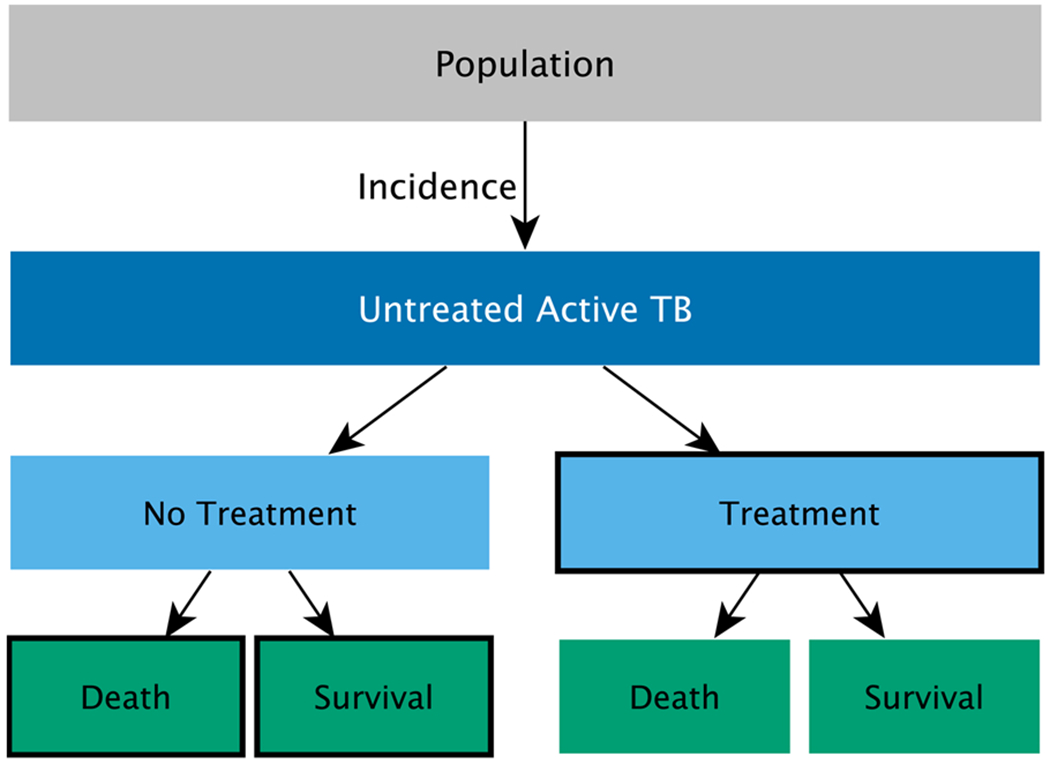
“Exits” from untreated active tuberculosis disease is used to approximate incidence rate. Boxes with black outlines indicate the three possible pathways. “Treatment” denotes TB cases initiating treatment and consequently notifying as a case in SINAN; “Death” denotes individuals who die from their disease prior to treatment initiation; “Survival” denotes individuals whose disease resolves prior to treatment initiation or death.

In this analysis, we estimated annual values for (i) the tuberculosis incidence rate and (ii) the fraction of individuals with tuberculosis who initiate treatment (“fraction treated”) for each Brazilian state and the Federal District (27 unique territories) over the period 2008–2017. Incidence is defined as the number of individuals in a population *developing* untreated active disease, and is infeasible to measure directly (Dye et al., 2008). Instead, we approximated incidence as the number of individuals *exiting* untreated active disease, which can be estimated in settings where TB treatment initiation and TB deaths are well documented. There are three possible pathways to exit the state of untreated active disease; individuals may initiate a TB treatment regimen, die before initiating treatment, or never initiate treatment and survive the disease (i.e. “self-cure”) (Fig. 1).

Information on the number of individuals exiting untreated active disease through TB treatment is available from the reported number of individuals initiating treatment (TB case notifications), minus individuals who have been misdiagnosed and individuals reentering treatment after previously being presumed lost to follow-up (treatment interrupted for more than 30 days). In Brazil, TB notification data are considered to provide an accurate measure of the total number of individuals initiating treatment, as treatment is exclusively available through the public healthcare system and clinicians are legally obligated to report TB cases. Duplicate notifications are routinely removed from the database using standard criteria (Oliveira et al., 2016).

Among individuals who develop active TB, a fraction will die before initiating treatment. Evidence on the number of individuals dying with active TB is available from official death records with TB as a primary or secondary cause of death. We approximated the total number of deaths among untreated individuals as the number of TB deaths observed in the death records minus the estimated number of TB fatalities among individuals receiving treatment, after adjusting for misreporting of TB as a cause of death and incomplete mortality system coverage. Finally, we assumed that the number of individuals who survive their disease without being treated (self-cure) is proportional to the number of deaths among individuals who do not receive treatment (Tiemersma et al., 2011).

### 2.2. Data

We accessed tuberculosis case records from 2008 to 2017 (n = 884 295) from the Brazil’s Notifiable Diseases Information System (SINAN; *Sistema de Informação de Agravos de Notificação)* (Ministério da Saúrde, 2007), a national reporting system for notifiable infectious diseases. Cases were spatially referenced based on the individual’s recorded municipality of residence at the time of their diagnosis. If there was no residence information recorded (n = 605; 0.07 %), we used the municipality where the individual received treatment as a proxy for residence. We excluded the small number of notifications where both fields were missing (n = 4; < 0.001 %). We also excluded notifications for individuals recorded as discontinuing treatment due to a misdiagnosis of TB (assumed to be TB negative; n = 19 414, 2.2 %), transfer patients (assumed to represent the continuation of an existing episode; n = 30 141, 3.4 %) or individuals reentering treatment after previously being presumed lost to follow-up (also assumed to represent the continuation of an existing episode; n = 61 561, 7.0 %). Finally, we excluded notifications for which the diagnosis was made post-mortem (n = 2 344; 0.27 %), as these individuals died prior to treatment initiation.

We accessed mortality data from 2008 to 2017 (n = 75 101) from the Brazilian Mortality Information System (SIM; *Sistema de Informação de Mortalidade*) (Ministério da Saúde, 2019). An individual was counted as having died with active TB if their death record contained at least one of the following International Classification of Disease (ICD-10) codes as a primary or secondary cause: A15.0–A19.9, K67.3, K93.0, M49.0, N74.0-N74.1, P37.0, or B20.0. These codes include records for TB-HIV deaths (World Health Organization, 2016). Deaths were spatially referenced based on the individual’s municipality of residence at the time of their death. We adjusted TB death counts for underreporting of TB as a cause of death (see details in Appendix 2) and for the estimated completeness of the mortality system (Queiroz et al., 2017; Schmertmann and Gonzaga, 2018).

There is little recent evidence on the fraction of TB cases that will self-cure in the absence of treatment, though historical data suggest approximately half will do so (Tiemersma et al., 2011). We conducted a survey of experts to generate an estimate for the fraction of individuals who self-cure prior to treatment initiation or death in Brazil (Appendix 2). We assumed that this value is proportional to the fraction of TB-related deaths that occur among individuals who do not initiate treatment.

We extracted data on state-level sociodemographic covariates that may be associated with tuberculosis incidence and case detection. We expect poverty to be positively associated with TB incidence (Harling et al., 2017); therefore, we included GDP per capita (gross domestic product per capita by municipality, averaged to the state-level). Additionally, healthcare access is a key determinant of TB diagnosis (Chin and Hanson, 2017). As it is difficult to measure primary care access, we considered coverage of the Family Health Strategy as a proxy. The Family Health Strategy is Brazil’s mechanism of primary care delivery; integrated care is provided by interdisciplinary Family Heath Teams who are responsible for the population of a defined geographic area (Andrade et al., 2018). We obtained official population estimates for each year and territory from the Brazilian Institute of Geography and Statistics (*Instituto Brasileiro de Geografia e Estatistica*, IBGE) (Instituto Brasileiro De Geografia E Estatística, 2018).

All data were de-identified and extracted from publicly available sources (Table 1).

### 2.3. Model structure

We specified Poisson likelihood functions for SINAN treatment notifications data and SIM TB mortality data for each territory (*i*) and year (*j*):

*Treatment Notifications_ij_* ~ *Poisson*( γ_ij_* α_ij_* *β_ij_*)

*TB Mortality_ij_* ~ *Poisson*(γ_ij_* α_ij_* [(β_ij_*δ_ij_) + (1 − *β_ij_*)*(1 − *μ*)]* π_i_* *ρ_ij_*)

In these functions *γ_ij_* represents the population size, *α_ij_* represents the TB incidence rate, *β_ij_* represents the fraction of cases initiating treatment, *δ_ij_* represents the probability of death among treated individuals, *μ* represents the probability of survival among untreated individuals, *π_i_* represents SIM coverage, and *ρ_ij_* represents an adjustment for the systematic misreporting of TB deaths in the SIM database.

Existing evidence on *δ_ij_*,*μ*, and *ρ_ij_* was summarized as prior probability distributions. We specified exponential and inverse logit functions for incidence (*α_ij_*) and fraction treated (*β_ij_*), respectively. These functions included a constant, a state-time random effect (allowed to follow a random walk over the study period), as well as FHS coverage and logtransformed GDP per capita as covariates. A full description of the model can be found in Appendix 1.

Using the fitted model, we estimated the rate of untreated TB cases for each territory and year, calculated as the modeled incidence multiplied by (1 – fraction treated). We also estimated the number of deaths before treatment initiation, calculated as the modeled number of TB deaths minus the modeled number of deaths occurring after treatment initiation. National-level estimates for TB incidence and untreated TB were obtained by summing state-level results. National-level estimates for the fraction treated were calculated as 1 minus the ratio of untreated TB to TB incidence at the national level.

The model was implemented using Stan and the rStan package for R (Stan Development Team, 2018a), which allows for the specification and estimation of Bayesian models (Stan Development Team, 2018b). We generated 4 000 samples from the posterior distributions of each quantity of interest; point estimates were calculated as the mean of this distribution, and uncertainty bounds were calculated as the 97.5 and 2.5 percentiles of this distribution. Model performance metrics and parameter posterior distributions can be found in Appendix 3.

Preliminary findings and final analyses were shared and discussed with collaborators in the Chronic and Airborne Disease Surveillance Coordination at the Brazilian Ministry of Health, via monthly phone calls and at two in-person meetings in Brazil. Our collaborators interpreted findings and assessed the validity of model assumptions and results against local programmatic knowledge.

This study description complies with the Guidelines for Accurate and Transparent Health Estimates Reporting (Stevens et al., 2016).

## 3. Results

### 3.1. Empirical observations

Between 2008 and 2017, the national rate of TB treatment initiations (TB case records minus post-mortem diagnoses, misdiagnoses of TB, transfers, and individuals reentering treatment after presumed loss to follow-up) decreased from 41.3–38.3 per 100 000, at an average annual rate of decline of 0.8 %. Over this period, the national TB death rate decreased from 4.1–3.6 deaths per 100 000 individuals in Brazil, at an average annual rate of 1.3 %.

### 3.2. Model results: 2017 estimates

For 2017, we estimated there were 41.5 TB cases per 100 000 (95 % uncertainty interval: 40.7, 42.5), and 4.8 (4.5, 5.0) TB deaths, for an average case-fatality of 11.5 % (11.0 %, 11.9 %). We estimate that there were 6 947 (5 348, 9 158) untreated cases of TB and 3 726 (3 261, 4 185) individuals who died from their disease before initiating treatment (Fig. S1).

**Fig. 2.**
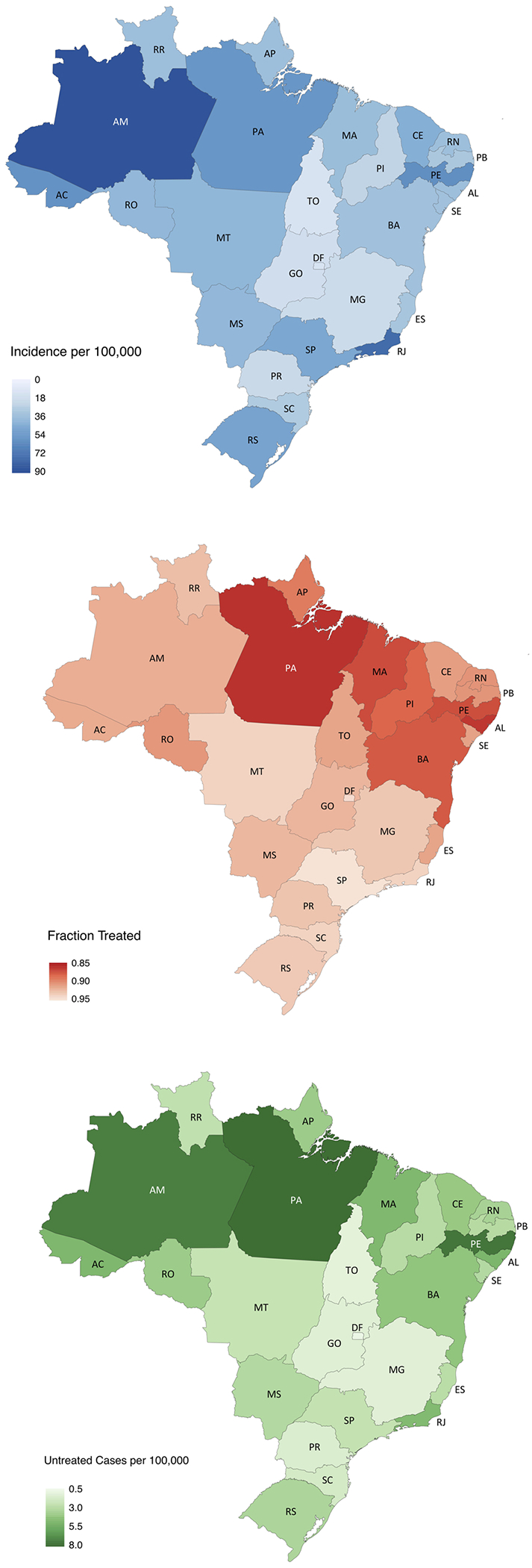
Tuberculosis burden estimates, Brazil 2017. Top: Modeled TB incidence per 100 000 individuals. Middle: Modeled fraction initiating treatment. Bottom: Modeled number of untreated cases per 100,000 individuals. A two-letter state code key can be found in Appendix 4.

Incidence rates for 2017 varied greatly by state. The highest modeled incidence rates were in Amazonas (88.3 cases per 100 000; 84.6, 92.4), Rio de Janeiro (77.0; 75.1, 79.5), and Pernambuco (59.6; 57.1, 62.6). The lowest modeled incidence rates were in the Federal District (11.7 cases per 100 000; 10.8, 12.8), Tocantins (12.2; 11.0, 13.5), and Goiás (16.5; 15.6, 17.6) (Fig. 2).

We estimated that 91.9 % (89.6 %, 93.7 %) of individuals with incident TB received treatment in 2017. São Paulo had the highest fraction treated (94.8 %; 92.4 %, 96.7 %) and Pará had the lowest (86.0 %; 81.4 %, 89.5 %). The fraction of cases treated generally followed a regional pattern; on average, states in the North and Northeast regions had a smaller fraction treated than states in the South, Southeast, and Center-West (Fig. 2; regional composition described in Appendix 3).

**Fig. 3.**
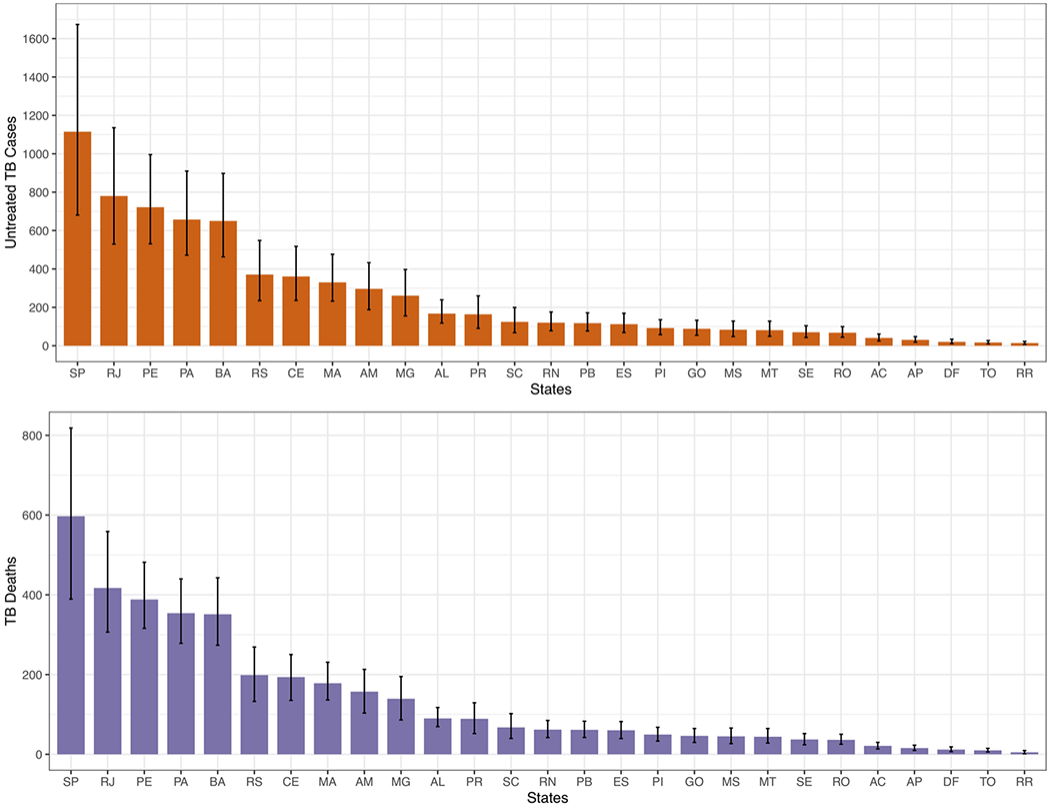
Untreated TB cases by state, Brazil, 2017. (Top) Untreated cases, calculated as the modeled number of incident TB cases multiplied by (1 – fraction treated); (Bottom) Deaths before treatment initiation, calculated as the modeled number of TB deaths minus the modeled number of TB deaths after treatment initiation. A two-letter state code key can be found in Appendix 3.

Nationally, we estimated that there were 3.3 untreated cases per 100 000 (2.6, 4.4) in 2017. Pará had the highest rate of untreated cases (7.8 per 100 000; 5.6, 10.8), and the Federal District had the lowest (0.7; 0.4, 1.1) (Fig. 2). The states of São Paulo and Rio de Janeiro had the largest number of untreated cases and deaths prior to treatment initiation (Fig. 3).

### 3.3. Model results: 10-year time trend

**Fig. 4.**
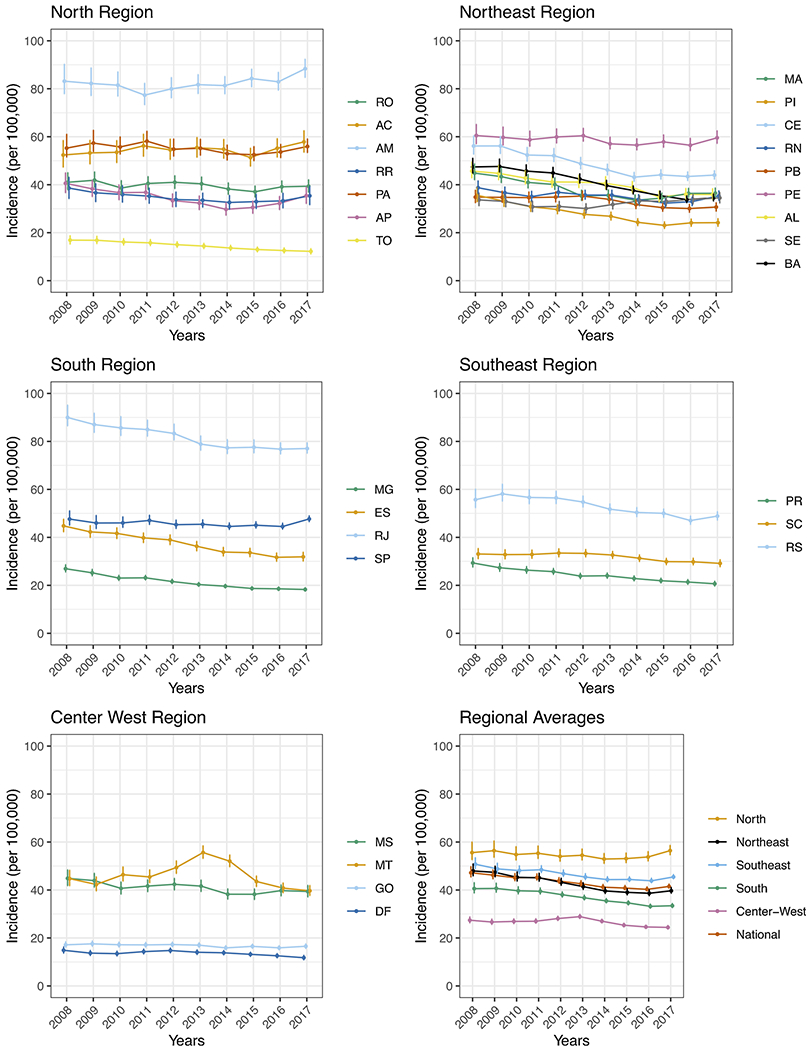
Modeled incidence, by state in Brazil, 2008–2017. Average annual change over the period, regional breakdown, and two-letter codes can be found in Appendix 3.

Over the period, we estimated that incidence decreased from 47.2 (45.3, 49.9) to 41.5 (40.7, 42.6) cases per 100 000 nationally, with an average annual rate of decline of 1.4 % (1.1 %, 1.9 %) (Fig. 4). The rate of decline in incidence rates varied by region, with the Southeast seeing the greatest reductions, at an average annual rate of 2.1 % (1.7, 2.5), and the North experiencing a modest increase, at an average annual rate of increase of 0.2 % (−0.5 %, 0.6 %) (Fig. 4). The average annual rate of decrease in incidence was largest in Minas Gerais (4.2 %; 3.6 %, 4.8 %) and Piauí (4.2 %; 3.2 %, 5.2 %); the average annual rate of decrease was < 0.1 % in Acre, Sergipe, Amazonas, Pará and São Paulo (Fig. 4).

Over the course of the study period, we estimated that the national fraction treated improved slightly, at an average annual rate of 0.56 % (0.22 %, 1.0 %). Similarly, most regions had a modest rate of improvement ranging from 0.31 % (−0.13 %, 0.90 %) in the North to 0.91 % (0.45 %, 1.5 %) in the South. The fraction treated increased the most in Rio Grande do Sul (average annual rate of 1.1 %; 0.6 %, 1.8 %), Mato Grosso (0.9 %; 0.4 %, 1.6 %), and Acre (0.8 %; 0.1 %, 1.7 %).

There were an estimated 85 810 (64 122, 115 887) untreated cases of TB in Brazil over the 10-year study period. The annual number of untreated cases decreased from an estimated 11 289 untreated cases (7 818, 16 330) in 2008 to 6 947 untreated cases (5 348, 9 158) in 2017. Nationally, the rate of untreated cases decreased from 6.0 (4.1, 8.6) to 3.4 (2.6, 4.4) cases per 100 000 over the study period. The largest average annual rate of decline in untreated cases was in the South region (average annual decrease of 9.9 %; 6.0 %, 13.4 %); the North region experienced the most modest decrease, with the uncertainty interval crossing zero (average annual rate of change of −1.9 %; −5.4 %, 1.8 %).

Additional results for individual states are provided in Appendix 3.

## 4. Discussion

Using a novel analytic approach, we estimated TB incidence and the fraction of cases initiating treatment from 2008 to 2017 for Brazil’s 26 states and Federal District. These results demonstrate progressive reductions in TB burden in Brazil over the 10-year study period, with TB incidence declining nationally at an average annual rate of A1.4 and the fraction of case initiating treatment increasing modestly. These estimates also reveal substantial subnational heterogeneity in TB burden and the strength of case detection, with incidence rates differing by a ratio of 7.5 to 1 (6.8, 8.2) between highest and lowest incidence states in 2017. The fraction treated and the rate of untreated cases followed a regional pattern; on average, poorer states in the North and Northeast had a smaller fraction treated and larger rate of untreated cases than wealthier states in the South and Southeast.

In the absence of data describing the development of TB disease, this analysis approximated TB incidence by quantifying exits from the disease state. For this reason, while our results should provide a valid measure of treatment coverage, they represent a slightly lagged estimate of ’true’ disease incidence. Moreover, this analysis relied heavily on the availability and validity of routinely-reported data on TB cases and deaths. Contemporary measures of the fraction of individuals with incident TB who self-cure without treatment do not exist; the most recent natural history study of TB outcomes in the absence of treatment was completed in 1970 (Tiemersma et al., 2011). Furthermore, there is limited evidence regarding under-reporting of TB as a cause of death; the only recent study of TB death under-reporting in Brazil was conducted in the city of Rio de Janeiro (de Oliveira Rocha et al., 2015) and is not generalizable to the rest of the country. To address these two limitations, we used an expert opinion survey to establish ranges of plausible values for these two quantities (Appendix 2).

In this study, we could have more accurately estimated the fatality rate among individuals receiving treatment had a linkage of SIM and SINAN been available. In the current analysis, we approximated this value as the fraction of cases whose treatment outcome was death, plus a modeled probability of mortality among individuals who were lost to follow-up. Furthermore, a linkage of the two systems would identify individuals with TB as a cause of death in SIM who did not appear in SINAN. In the absence of a linkage study, we made the simplifying assumption that the difference between expected deaths in SINAN and SIM death counts (after adjusting for under-reporting) was due to deaths among individuals who had not been notified in SINAN. This limitation is applicable to all studies conducted in settings where a robust linkage of disease notification and mortality data systems has not been performed. Finally, SINAN provides information about individuals receiving treatment, which may be less than the number of individuals with TB who receive a diagnosis. In settings where there are low rates of primary default, the fraction treated will approximate the case detection rate.

Reliable methods for small area incidence estimation are central to efforts to improve case finding activities, particularly in countries with decentralized healthcare systems. The WHO has developed a number of methods to assess and improve the quality of TB burden estimates (Glaziou et al., 2017). Inventory studies leverage data from nationally representative surveys of tuberculosis prevalence, and so provide independent estimates of disease burden unaffected by local differences in case detection. However, inventory studies are expensive and logistically difficult to implement (Glaziou et al., 2012). Other recently reported approaches for subnational estimation utilize existing estimates of case detection rates, which may not always be available (Avilov et al., 2014); furthermore, existing estimates of subnational tuberculosis trends in Brazil are produced by applying a smoothing model to TB case notification data, and do not account for undiagnosed or untreated TB (Ross et al., 2018). In contrast, our model does not require assumptions about case detection or national incidence rates, quantifies the fraction of cases that receive treatment, and relies on routinely collected data and expert opinion only. Expert opinion is a reasonable source for parameters that are difficult to quantify, and the inherent uncertainty can be easily propagated in the model through the use of weakly informative prior distributions. The data required to parametrize the model are relatively easy to collect, and the model can be easily implemented in other settings with disaggregated case notification and mortality data.

## 5. Conclusion

Undetected and untreated tuberculosis is a major public health problem. Reliable subnational TB burden estimates enable health authorities to identify areas of low case detection. We present a novel estimation approach that uses routinely collected data to produce longitudinal estimates of the TB incidence rate and fraction treated. Using data from Brazil, we described trends in TB incidence and identified states with low fractions of treated cases. The approach we describe may be applicable in other settings with compulsory TB case notification and reasonably accurate and complete death records.

## Supplementary Material

1

2

## Figures and Tables

**Table 1:** Data description and sources.

Variable	Description	Data Source
TB Treatment Initiations	TB case records, minus post-mortem diagnoses, misdiagnoses of TB, and individuals reentering treatment after presumed loss to follow-up, by territory and year.	SINAN-TB Oliveira et al. 2016
TB Deaths	Number of deaths with a TB-related ICD-10 code as a primary or secondary cause, by territory and year.	SIM Ministério da Saúde (2019)
TB Deaths After Notification in SINAN	Notified TB cases with “death” as the recorded treatment outcome.	SINAN-TB
Population	Population estimates by territory and year.	Brazilian Institute of Geography and Statistics (IBGE) Instituto Brasileiro De Geografia E Estatística 2018
Primary Care Access	Number of Family Health Teams per 4 000 people, by territory and year. One team per 4 000 population represents target coverage level; (Andrade et al., 2018) if coverage was greater than 100 %, territories were assigned a value of 100 %.	Health Informatics, Brazilian Ministry of Health (DATASUS) Ministério da Saúde 2018
GDP Per Capita	Economic value of goods produced, by territory and year.	Brazilian Institute of Geography and Statistics (IBGE)
Deaths from a Poorly Defined Cause	Percentage of deaths for which the primary cause of death is listed with a chapter XVIII ICD-10 code by territory and year.	Health Informatics, Brazilian Ministry of Health (DATASUS)
Mortality System Coverage	Estimate for the fraction of total deaths that are recorded in SIM, by territory.	Queiroz et al. (2017), Schmertmann and Gonzaga 2018
