# Supplementary material for: Bayesian evidence synthesis to estimate subnational TB incidence: An application in Brazil
Source: Epidemics. Author manuscript; Available in PMC 2021 Jul 2. (PMC8252152; doi:10.1016/j.epidem.2021.100443)
Supplement: 2 [file NIHMS1715755-supplement-2.docx]

**Appendix 1: Model Description**

The expected number of cases initiating treatment in a state was modeled as the product of true incidence and the fraction of treated cases. The expected number of TB deaths reported in SIM was modelled as a function of incidence, the probability of death among treated cases, the probability of death for cases that did not receive treatment, and an adjustment factor for SIM coverage and systematic under-reporting of TB deaths. We specified Poisson likelihood functions for SINAN case notification data and SIM mortality data. Estimation was performed simultaneously for all geographies and time points. The overall likelihood was calculated as the product of all component likelihoods, assuming independence of all data conditional on the model parameters (essentially, assuming no unmodelled systematic bias in the observed data):

$$Case Notifications_{ij} \sim Poisson( \gamma_{ij}* \alpha_{ij}* \beta_{ij})$$

$$TB Mortality_{ij} \sim Poisson\left( \gamma_{ij}* \alpha_{ij}*\left[ \left( \beta_{ij}*\frac{A_{ij}+\delta B_{ij}}{C_{ij}-D_{ij}} \right)+\left( \left( 1- \beta_{ij} \right)*\left( 1-\mu\right) \right) \right]* \pi_{i}* \rho_{ij} \right)$$

For territory *i* in year *j*; where $\gamma_{ij}$represents observed population size, $\alpha_{ij}$ represents modeled incidence rate, $\beta_{ij}$ represents the fraction of treated cases, $\delta$represents the probability of death given an individual was lost to follow-up, $\mu$ represents the estimated probability of survival without treatment, $\pi_{i}$ represents the estimated SIM coverage, *A_ij_* is a vector of observed deaths on treatment, *B_ij_* is a vector of observed cases lost to follow-up, *C_ij_* is a vector of observed TB cases that received treatment, *D_ij_* is a vector of observed TB cases for which no treatment outcome was recorded. *A_ij_*, *B_ij_*, *C_ij_*, and *D_ij_* are observed quantities which indicate the fraction of individuals who die with active tuberculosis after treatment initiation. Finally, $\rho_{ij}$ represents an adjustment for the systematic misreporting of TB deaths in the SIM database:

$$Death Adjustment_{ij}=\rho_{ij}=logit^{-1}( \theta_{0}+ \theta_{1i}*\sigma_{\rho}+\theta_{2}*( j-10)+\theta_{3}x_{ij})$$

For state *i* in year *j*; where $\theta_{0}$ is the intercept, $\theta_{1}*\sigma$is a state-level random effect, $\theta_{2}*( j-10)$ is a linear time trend (beginning at the final year of the time series), $x_{ij}$ represents the percentage of deaths in SIM attributed to a poorly-defined cause, and $\theta_{3}$ is the associated regression coefficient. Expert opinion was used to determine appropriate prior distributions for $\theta_{3}$, described in the next section.

We specified exponential and inverse logit functions for incidence and fraction detected, respectively:

$$Incidence_{i}=\alpha_{i}=exp \left( \varphi_{0}+ {\varphi_{1}}_{ij}+ {\varphi X}_{ij} \right)$$

$$Fraction {Detected}_{i}=\beta_{i} =logit^{-1}\left( \omega_{0}+ {\omega_{1}}_{ij}+ {\omega X}_{ij} \right)$$

For state *i* at time *j*; where $\varphi_{0}$ and $\omega_{0}$are constants; $X_{ij}$ is a vector of state-level covariates including primary care access and average GDP per capita; and $\varphi$ and $\omega$ are the associated vectors of regression coefficients. The inclusion of these variables allows for partial pooling among states with similar sociodemographic characteristics. Additionally, ${\varphi_{1}}_{ij}$ and ${\omega_{1}}_{ij}$ are state-time random effects:

$${\varphi_{1}}_{ij}= \psi_{1,i}+ \psi_{2,ij-1}$$

$${\omega_{1}}_{ij}=\phi_{1,i}+ \phi_{2,ij-1}$$

For state *i* in time *j*; where $\psi_{1,i}$ and $\phi_{1,i}$ are demeaned state-level random effects, and $\psi_{2,ij-1}$ and $\phi_{2,ij-1}$ are demeaned year effects, allowed to follow a random walk. All parameters were assigned normal priors with a mean of zero and a variance with a half-Cauchy prior (see Appendix 3).

*Prior Distributions Specification*

There is substantial uncertainty around true values for several model parameters. We used a Bayesian approach to represent and propagate this uncertainty through the analysis. The prior distributions for misreporting of TB deaths and for the case fatality of untreated TB were elicited through an expert opinion survey, described in Appendix 2. Prior distributions for all other model parameters were chosen to be weakly informative, in order to allow the model to be fit with limited external influence while still excluding implausible values (Table S1).

R code for the model may be made available upon written request to the corresponding author. The model ran for 3500 iterations on four chains with a burn-in of 2500 iterations. The total run time was approximately 10 minutes. There were no divergent transitions or iterations that saturated the maximum tree depth of 14.

*Table S1:* Prior and Posterior Distributions for Key Model Parameters

| Parameter | Prior Distribution | Posterior | | | | |
| --- | --- | --- | --- | --- | --- | --- |
|  |  | Mean | Lower Bound (2.5%) | Upper Bound (97.5%) | Effective Sample Size | R.hat |
| ϕ_0_ | Normal (0, 10) | 3.5992 | 3.5739 | 3.6336 | 873.0402 | 1.0067 |
| ϕ_GDP_ | Normal (0, 10) | -0.0727 | -0.1164 | -0.0315 | 697.4057 | 1.0050 |
| ϕ_FHS_ | Normal (0, 10) | -0.0785 | -0.1293 | -0.0266 | 708.1051 | 1.0038 |
| σ_ϕi0_ | Cauchy* (0, 2) | 0.4534 | 0.3420 | 0.6050 | 3572.0927 | 1.0000 |
| σ_ϕij_ | Cauchy* (0, 2) | 0.0494 | 0.0427 | 0.0566 | 1464.0860 | 0.9996 |
| ω_0_ | Normal (0, 10) | 2.2100 | 1.9182 | 2.4726 | 880.5324 | 1.0070 |
| ω_FHS_ | Normal (0, 10) | 0.0958 | -0.0347 | 0.2213 | 822.4305 | 1.0029 |
| ω_GDP_ | Normal (0, 10) | 0.2924 | 0.1511 | 0.4254 | 724.6488 | 1.0040 |
| σ_ωi0_ | Cauchy* (0, 2) | 0.1135 | 0.0042 | 0.2948 | 47.8056 | 1.0773 |
| σ_ωij_ | Cauchy* (0, 2) | 0.1290 | 0.1015 | 0.1623 | 471.4899 | 1.0075 |
| θ_0_ | Normal (0, 1) | -1.6805 | -1.9800 | -1.3955 | 1020.2546 | 1.0076 |
| θ_2_ | Normal (0, 0.05) | -0.0734 | -0.1182 | -0.0251 | 612.3188 | 1.0037 |
| θ_3_ | Normal (0, 1) | 2.8283 | 1.3593 | 4.2563 | 4649.7348 | 0.9993 |
| σ_ρ_ | Cauchy* (0, 2) | 0.5672 | 0.2960 | 0.9108 | 378.5527 | 1.0157 |
| μ | Beta (25.65, 33.32) | 0.4523 | 0.3256 | 0.5824 | 1483.4679 | 1.0027 |
| τ | Beta (4.29, 81.47) | 0.0349 | 0.0106 | 0.0716 | 1938.5318 | 1.0003 |
| A | Beta (52.97, 451.2) | 0.1581 | 0.1332 | 0.1850 | 2238.0554 | 1.0017 |
| B | Beta (97.83, 285.8) | 0.2182 | 0.1854 | 0.2541 | 3039.5647 | 1.0017 |

*Cauchy distributions were implemented as half-cauchy (constrained at initialization with a lower bound of zero).

**Appendix 2: Expert Opinion Survey**

An expert opinion survey was circulated to individuals who are either Brazilian tuberculosis researchers or have recently published research relating to tuberculosis in Brazil. The survey design was adapted from Pill (1971).^1^ An initial survey was sent to a smaller group (n = 4) to determine appropriate ranges for survey value options. A second survey, adjusted based on results from the first, was circulated to a broader group (n = 9). A total of 8 responses were collected. Respondents included U.S.- and Brazil-based researchers and epidemiologists who conduct work related to tuberculosis surveillance in Brazil.

Expert survey respondents were asked to provide the highest possible, lowest possible, and best estimate values for TB death under-reporting given two scenarios in the year 2017. In both scenarios, respondents were asked to consider the number of TB deaths out of 100 that would be misclassified and therefore not appear in SIM with an ICD-10 code associated with TB. In Scenario A, we described a location where very few (1%) of total deaths have a poorly-defined cause. In Scenario B, we described a location where considerably more (15%) of total deaths have a poorly-defined cause. The survey was circulated in English and Portuguese. The median values from respondents can be found in Table 1. These values were then represented as a Beta distribution, which were used as the prior distribution for A and B:

*A* ~ Beta (52.97, 451.54)

*B* ~ Beta (97.83, 285.81)

The values A and B were then used to model the death adjustment as follows:

$A=logit^{-1}(\zeta_{1}+ \theta_{3}*y)$

$B=logit^{-1}(\zeta_{1}+ \theta_{3}*z)$

Where $\zeta_{1}$ is the average value for a state-level random effect ($\theta_{1}+ \theta_{2i}*\sigma)$ and *y* and *z* where given the prior distributions:

*y* ~ Normal (0.01, 0.001)

*z* ~ Normal (0.15, 0.001)

The prior distribution for self-cure was also determined using the expert survey. Respondents were asked to provide the highest possible, lowest possible, and best estimate values for long-term survival among individuals with active TB who are never diagnosed and never received treatment. Median values from respondents can be found in Table S2. These values were converted to a Beta distribution, which was used the prior distribution for the probability of survival given a case wasn’t notified:

*Survival | No Notification* = $\mu_{i}$~ Beta (25.65, 33.32)

Table S2: Median response values on expert opinion survey

|  | Lowest | Highest | Best Estimate |
| --- | --- | --- | --- |
| Scenario A | 4.5 | 21 | 10.5 |
| Scenario B | 19.5 | 46.5 | 25.2 |
| Self-Cure | 22.5 | 47.5 | 43.5 |

1 Pill J. The Delphi Method: substance, context, a critique, and an annotated bibliography. Socio-Econ Plan. Sci, 1971. **5:** 57-71

**Appendix 3: Additional Results**

*Table S3:* State-Level Estimates for 2017

| State | Two-Letter Code | Region | New Cases (per 100,000) | Incidence (per 100,000) | Fraction Treated | Untreated Cases (per 100,000) |
| --- | --- | --- | --- | --- | --- | --- |
| Rondônia | RO | North | 36.0 | 39.4 (36.7, 42.2) | 0.91 (0.87, 0.94) | 67.6 (44.2, 99.4) |
| Acre | AC | North | 56.0 | 57.9 (53.5, 62.6) | 0.92 (0.88, 0.95) | 40.3 (24.6, 60.9) |
| Amazonas | AM | North | 81.7 | 88.3 (84.6, 92.4) | 0.92 (0.88, 0.95) | 296.0 (187.7, 432.6) |
| Roraima | RR | North | 38.1 | 35.4 (31.6, 39.4) | 0.93 (0.89, 0.96) | 13.6 (7.0, 22.3) |
| Pará | PA | North | 48.2 | 55.9 (53.3, 59.2) | 0.86 (0.81, 0.89) | 657.1 (471.8, 909.9) |
| Amapá | AP | North | 33.1 | 35.4 (32.1, 39.1) | 0.89 (0.84, 0.93) | 30.3 (18.4, 47.4) |
| Tocantins | TO | North | 10.6 | 12.2 (11.0, 13.5) | 0.91 (0.86, 0.95) | 16.7 (9.7, 27.0) |
| Maranhão | MA | Northeast | 31.7 | 36.4 (34.6, 38.8) | 0.87 (0.82, 0.91) | 330.0 (232.1, 476.5) |
| Piauí | PI | Northeast | 21.4 | 24.2 (22.5, 26.0) | 0.88 (0.83, 0.92) | 92.1 (57.7, 135.4) |
| Ceará | CE | Northeast | 40.2 | 44.0 (42.2, 46.0) | 0.91 (0.87, 0.94) | 360.7 (236.1, 517.5) |
| Rio Grande do Norte | RN | Northeast | 33.0 | 35.4 (33.3, 37.6) | 0.90 (0.86, 0.94) | 119.8 (77.7, 175.8) |
| Paraíba | PB | Northeast | 28.4 | 30.7 (28.8, 32.6) | 0.90 (0.87, 0.94) | 117.3 (76.4, 171.3) |
| Pernambuco | PE | Northeast | 52.1 | 59.6 (57.1, 62.6) | 0.87 (0.83, 0.90) | 721.4 (530.9, 995.7) |
| Alagoas | AL | Northeast | 31.0 | 35.9 (33.7, 38.5) | 0.86 (0.81, 0.90) | 167.1 (117.7, 239.2) |
| Sergipe | SE | Northeast | 31.9 | 34.5 (32.2, 36.9) | 0.91 (0.87, 0.94) | 70.0 (42.8, 104.1) |
| Bahia | BA | Northeast | 30.2 | 34.5 (33.1, 36.3) | 0.88 (0.84, 0.91) | 649.7 (463.0, 897.7) |
| Minas Gerais | MG | Southeast | 17.1 | 18.3 (17.6, 19.1) | 0.93 (0.90, 0.96) | 260.5 (155.2, 397.2) |
| Espírito Santo | ES | Southeast | 29.2 | 31.9 (30.1, 33.8) | 0.91 (0.87, 0.94) | 112.2 (68.7, 169.1) |
| Rio de Janeiro | RJ | Southeast | 72.7 | 77.0 (75.1, 79.5) | 0.94 (0.91, 0.96) | 779.9 (529.3, 1135.7) |
| São Paulo | SP | Southeast | 45.3 | 47.7 (46.5, 49.0) | 0.95 (0.92, 0.97) | 1114.6 (680.6, 1673.6) |
| Paraná | PR | South | 19.1 | 20.7 (19.7, 21.7) | 0.93 (0.89, 0.96) | 163.6 (90.7, 259.8) |
| Santa Catarina | SC | South | 27.3 | 29.2 (27.7, 30.7) | 0.94 (0.91, 0.97) | 124.0 (67.9, 199.2) |
| Rio Grande do Sul | RS | South | 45.7 | 48.9 (47.2, 50.7) | 0.93 (0.90, 0.96) | 370.6 (235.0, 548.4) |
| Mato Grosso do Sul | MS | Center-West | 36.3 | 39.4 (37.1, 42.0) | 0.92 (0.88, 0.95) | 83.1 (47.8, 128.5) |
| Mato Grosso | MT | Center-West | 37.1 | 39.7 (37.7, 41.9) | 0.94 (0.91, 0.96) | 80.7 (49.3, 128.1) |
| Goiás | GO | Center-West | 15.5 | 16.5 (15.6, 17.6) | 0.92 (0.89, 0.95) | 88.2 (54.6, 132.6) |
| Distrito Federal | DF | Center-West | 10.7 | 11.8 (10.8, 12.8) | 0.94 (0.91, 0.97) | 19.8 (11.0, 33.9) |

*Table S4:* Modeled Average Annual Changes in State-Level TB Burden, 2008 - 2017

| State | Two-Letter Code | Region | Incidence (per 100,000) | Fraction Treated | Untreated Cases (per 100,000) |
| --- | --- | --- | --- | --- | --- |
| Rondônia | RO | North | -0.45 (-1.6, 0.6) | 0.579 (-0.08, 1.4) | -4.6 (-9.6, 0.9) |
| Acre | AC | North | 1.12 (-0.3, 2.5) | 0.776 (0.05, 1.7) | -4.7 (-10.5, 1.1) |
| Amazonas | AM | North | 0.68 (-0.2, 1.4) | 0.594 (0.04, 1.3) | -4.2 (-8.7, 0.7) |
| Roraima | RR | North | -0.96 (-2.7, 0.8) | 0.565 (-0.14, 1.4) | -6.1 (-12.8, 1.0) |
| Pará | PA | North | 0.14 (-0.7, 0.8) | -0.008 (-0.57, 0.7) | 0.5 (-3.9, 5.3) |
| Amapá | AP | North | -1.50 (-2.9, -0.1) | -0.046 (-0.71, 0.6) | -1.0 (-6.8, 5.5) |
| Tocantins | TO | North | -3.53 (-5.0, -2.0) | 0.558 (-0.23, 1.5) | -7.8 (-14.0, -1.1) |
| Maranhão | MA | Northeast | -2.27 (-3.0, -1.6) | 0.542 (0.03, 1.2) | -5.2 (-8.3, -2.1) |
| Piauí | PI | Northeast | -4.20 (-5.2, -3.2) | 0.542 (-0.16, 1.4) | -7.3 (-11.9, -2.6) |
| Ceará | CE | Northeast | -2.66 (-3.3, -2.0) | 0.712 (0.24, 1.3) | -7.7 (-10.9, -4.3) |
| Rio Grande do Norte | RN | Northeast | -1.01 (-1.9, -0.2) | 0.209 (-0.32, 0.8) | -2.7 (-7.3, 2.4) |
| Paraíba | PB | Northeast | -1.39 (-2.3, -0.5) | 0.605 (0.01, 1.3) | -5.7 (-9.8, -1.0) |
| Pernambuco | PE | Northeast | -0.17 (-0.8, 0.4) | 0.260 (-0.21, 0.8) | -1.7 (-5.1, 1.8) |
| Alagoas | AL | Northeast | -2.65 (-3.5, -1.8) | -0.174 (-0.73, 0.4) | -1.4 (-5.6, 3.3) |
| Sergipe | SE | Northeast | 0.27 (-0.8, 1.3) | 0.312 (-0.25, 1.0) | -2.4 (-7.7, 3.1) |
| Bahia | BA | Northeast | -3.47 (-4.1, -2.9) | 0.201 (-0.23, 0.8) | -4.6 (-7.9, -1.4) |
| Minas Gerais | MG | Southeast | -4.21 (-4.9, -3.6) | 0.628 (0.14, 1.2) | -10.0 (-14.5, -5.2) |
| Espírito Santo | ES | Southeast | -3.70 (-4.5, -2.9) | -0.009 (-0.46, 0.5) | -3.5 (-8.5, 1.7) |
| Rio de Janeiro | RJ | Southeast | -1.71 (-2.1, -1.3) | 0.516 (0.24, 0.9) | -7.3 (-10.1, -4.7) |
| São Paulo | SP | Southeast | 0.02 (-0.7, 0.5) | 0.755 (0.27, 1.4) | -8.2 (-12.6, -3.4) |
| Paraná | PR | South | -3.82 (-4.6, -3.1) | 0.701 (0.10, 1.4) | -10.0 (-15.2, -4.3) |
| Santa Catarina | SC | South | -1.39 (-2.2, -0.6) | 0.584 (0.08, 1.2) | -7.6 (-12.9, -1.9) |
| Rio Grande do Sul | RS | South | -1.44 (-2.2, -0.8) | 1.127 (0.59, 1.8) | -10.3 (-14.0, -6.5) |
| Mato Grosso do Sul | MS | Center-West | -1.44 (-2.4, -0.5) | 0.304 (-0.28, 1.0) | -4.3 (-10.4, 2.1) |
| Mato Grosso | MT | Center-West | -1.32 (-2.2, -0.4) | 0.944 (0.43, 1.6) | -9.9 (-14.5, -5.3) |
| Goiás | GO | Center-West | -0.39 (-1.3, 0.5) | 0.411 (-0.12, 1.0) | -4.1 (-9.4, 1.2) |
| Distrito Federal | DF | Center-West | -2.56 (-3.8, -1.3) | 0.369 (-0.11, 0.9) | -7.2 (-13.5, -0.5) |

*Negative numbers indicate an average decrease in the metric.
